# Roles of Epithelial and Mesenchymal TRP Channels in Mediating Inflammatory Fibrosis

**DOI:** 10.3389/fimmu.2021.731674

**Published:** 2022-01-04

**Authors:** Yuka Okada, Takayoshi Sumioka, Peter S. Reinach, Masayasu Miyajima, Shizuya Saika

**Affiliations:** ^1^ Ophthalmology, Wakayama Medical University, Wakayama, Japan; ^2^ Wenzhou Medical University School of Ophthalmology and Optometry, Wenzhou, China

**Keywords:** TRP, cornea, wound healing, nurotrophic keratitis, fibrosis

## Abstract

The maintenance of normal vision is dependent on preserving corneal transparency. For this to occur, this tissue must remain avascular and its stromal architecture needs to be retained. Epithelial transparency is maintained provided the uppermost stratified layers of this tissue are composed of terminally differentiated non-keratinizing cells. In addition, it is essential that the underlying stromal connective tissue remains avascular and scar-free. Keratocytes are the source of fibroblasts that are interspersed within the collagenous framework and the extracellular matrix. In addition, there are sensory nerve fibers whose lineage is possibly either neural crest or mesenchymal. Corneal wound healing studies have been undertaken to delineate the underlying pathogenic responses that result in the development of opacification following chemical injury. An alkali burn is one type of injury that can result in severe and long- lasting losses in ocular transparency. During the subsequent wound healing process, numerous different proinflammatory cytokines and proteolytic enzymes undergo upregulation. Such increases in their expression levels induce maladaptive expression of sustained stromal inflammatory fibrosis, neovascularization, and losses in the smooth optical properties of the corneal outer surface. It is becoming apparent that different transient receptor potential channel (TRP) isoforms are important players in mediating these different events underlying the wound healing process since injury upregulates both their expression levels and functional involvement. In this review, we focus on the involvement of TRPV1, TRPA1 and TRPV4 in mediating some of the responses that underlie the control of anterior ocular tissue homeostasis under normal and pathological conditions. They are expressed on both different cell types throughout this tissue and also on corneal sensory nerve endings. Their roles have been extensively studied as sensors and transducers of environmental stimuli resulting from exposure to intrinsic modulators and extrinsic ligands. These triggers include alteration of the ambient temperature and mechanical stress, etc., that can induce pathophysiological responses underlying losses in tissue transparency activated by wound healing in mice losses in tissue transparency. In this article, experimental findings are reviewed about the role of injury-induced TRP channel activation in mediating inflammatory fibrotic responses during wound healing in mice.

## Rationale for Focus on TRPV1, TRPA1 and TRPV4 Involvement in Ocular Biology

There is accumulating evidence that TRPV1, TRPA1 and TRPV4 channels may serve as targets to improve therapeutic management of chronic corneal injury and pain resulting from chemical injury (1). One of our interests entails characterizing their role in mediating responses to environmental challenges that include those encountered during wound healing. These studies are prompted by the realization that there is a lack of treatment options to deal with chemical burn symptomology in a clinical setting. Specifically, the current procedures have limited effectiveness in treating the inflammation and scarring, which detract from a favorable wound healing response. If these responses to injury are severe, the only therapeutic option may be surgical intervention, which also can have some limitations. The results of these studies have shown that each of channels are viable drug targets in animal and human studies

Initially it was hoped that a first generation of TRPA1, TRPV1 and TRPV4 antagonists could be developed for treating losses in corneal transparency resulting from inflammation and scarring induced by severe corneal injury. However, numerous animal and human screening has shown that their systemic administration induces hyperthermia alongside a risk of loss of awareness of hot surfaces. However, second generation TRPV1 antagonists are now reported to be in clinical trials that lack this hyperthermia effect (2). Despite these stumbling blocks in drug development, there are indications that continuance of studies on their roles in mediating responses to corneal injury may help design agents that selectively block injury-induced TRP channel activation.

## Characterization of TRP Channel Expression and Function

Immunohistochemistry and Western blot analysis are used to validate TRP channel presence and characterize their protein expression levels. The validity of these findings depends on using antibodies having adequate sensitivity and selectivity to accurately characterize their contribution to a particular phenotype. Prior to their use, their sensitivity and specificity are evaluated using cell lines transfected with the antigen that is expressed in a broad span of different human tissues. The antibodies that are relied on generate Western blots in which different investigators showed that their normalized expression levels were reproducible in knockdown experiments using different cell lines. Identifying their functional involvement in mediating responses to relevant environmental challenges and selective agonists and antagonists includes employing Ca^2+^ fluorescence cell imaging in combination with patch clamp technology. Their response patterns are used to validate the identity of a TRP channel subtype that responds to an environmental challenge. A cautionary note is warranted since sole reliance on functional blocking with an antagonist can be misleading. This is possible because its selectivity can be species specific. This limitation is evident since in preclinical animal studies an antagonist was effective whereas it had no inhibitory activity in humans (2).

## Expression Levels and Functional Heterogeneity of Transient Receptor Potential (TRP) Channels in the Cornea

The cornea is unique in that it contains the highest density of sensory neurons in the entire human body. They are richly endowed with functional thermosensitive TRPV1, TRPA1 and TRPM8 activity. These ionic channels are activated by both specific environmental challenges and injurious conditions at the ocular surface. In chemical injury models, an alkali burn induces corneal inflammation neovascularization and scarring along with sensory transduction leading to pain and losses in corneal transparency. As the treatment options can be limited to performing corneal keratoplasty, determining their involvement in mediating pathological responses has become an attractive option since treatment for the most part is limited to merely providing symptomatic relief in a clinical setting. These studies suggest that future studies are warranted to determine if these different TRP subtypes can serve as targets to improve therapeutic management of chemical injury in a clinical setting.

An archetype TRP channel gene was first cloned from a blind Drosophila mutant in 1989 (3). Sequence analysis revealed that its RNA transcript has a molecular sequence of 4.1kb that encodes a 1,275 amino acid protein (3, 4). This channel is unique because it does not have appreciable structural similarity with any other known proteins. Subsequent studies showed that this channel is indeed an archetype of a TRP channel superfamily that is composed of 7 heterogeneous subfamilies made up of 28 different isoforms in mammals. Many of these different subtypes have specific roles in mediating a host of responses to a wide variety of ambient stresses such as changes in temperature, membrane stretch, medium pH osmolality, and exposure to exogenous modulators of kinase-induced changes in TRP channel phosphorylation status (5–7). The vanilloid subfamily contains 6 different TRPV subtypes. The characteristics of the TRP Vanilloid 1 (TRPV1) isoform are better understood than those associated with others in the same subfamily. These subtypes are expressed throughout a wide variety of different human tissues including peripheral nerve endings, brain and the spinal cord. It is the predominant heat detector in peripheral sensory neurons that innervate tissues throughout the body. This subtype is cation permeable enabling Ca^2+^ influx that is enhanced above 42°C, exposure to a hypertonic challenge, mechanical stretch, a reduction in pH below 6.5, and by a variety of chemicals, including capsaicin, piperin, resinferatoxin, olvanil, zingerone. In addition, different types of tissue injury can induce increases in the expression level of agents that induce the release of cellular constituents that in turn stimulate TRPV1 activity. Two of these constituents include bradykinin and nerve growth factor (NGF). They both are released at sites of tissue injury and serve to increase both pain sensitivity as well as thresholds for inducing hyperalgesia (8). The satiety factor, oleoylethanolamide (OEA) can also activate TRPV1. Agents such as bradykinin and nerve growth factor (NGF) increase pain sensitivity thresholds that underlie hyperalgesia (8). However, this response is dependent on prior protein kinase C (PKC)-induced TRPV1 phosphorylation (9). Capsaicin is a relatively selective TRPV1 channel agonist and its ability to induce Ca^2+^ transients is used to determine functional TRPV1 expression. A model for how capsaicin selectively activates this channel was derived based on extensive investigation using a variety of experimental techniques including mutagenesis, patch-clamp recording, crystallography, cryo-electron microscopy, computational docking and molecular dynamic simulation (10). Stromal fibroblasts express TRPV1 activity that is confirmed based on accompanying rises in underlying ionic currents (11). These capsaicin-induced responses precede increases in proinflammatory cytokine release along with increases in α-smooth muscle expression that induce myofibroblast transdifferentiation and other phenotypic changes.

TRPA1 is one of the 28 different TRP subtypes that belongs to the ankyrin subfamily. It is a broadly selective thermosensitive cation channel activated by allyl isothiocyanate, AITC, a selective agonist, which facilitates increases in intracellular Ca^++^, Na ^+^ and K^+^ influx. It has been used as a model substance to characterize TRPA1 since it is one of the most efficient activators of TRPA1 (12). Its activation is responsible for the pungent and lachymatory effects of mustard, horseradish, and wasabi (13). TRPA1 selectivity has been validated in numerous animal and human derived tissues. AITC likely activates TRPA1 through a mechanism involving direct covalent modification of specific cysteine residues within the channel protein.

This TRP subtype is present on both corneal nerves and on corneal epithelial and endothelial cells as well as stromal fibroblasts and macrophages that are induced to infiltrate into the stroma in a corneal wound injury model. It is also expressed on sensory neurons arising from the trigeminal, dorsal root and nodose ganglia as well as a subpopulation of Aδ- and C-fiber nociceptive sensory neurons. TRPA1 responds to several pungent compounds such as isothiocyanates, allicin and cinnamaldehyde. There is suggestive evidence that TRPA1 activation may contribute to inducing skin allodynia. This condition develops during inflammation of the skin and it plays an important role in eliciting histamine-insensitive itch neurons. TRPA1 is activated at temperatures that fall below 17°C. This channel also serves as mechano-sensitive and pain sensor mediator. Several lines of evidence suggest that TRPA1 and TRPV1 mutually elicit inflammation-induced noxious stimuli in sensory neurons (14). TRPV1activation in turn alters the underlying mechanisms controlling TRPA1 channel activation. Such crosstalk is applicable to our studies in which we identified a dependence of TGFβ-1 driven fibrosis and inflammation on TRPV1 activation. Another indication that crosstalk has physiological relevance stems from our study in which it was shown that TGF-β induced increases in inflammatory fibrosis are also dependent on TRPA1 activation (15).

TRPV4 ion channels are activated by a diverse array of biochemical and biomechanical stimuli including mechanical deformation osmotic stimuli, 27 to 35°C cannabinoids, and arachidonic acid. These Ca^2+^-permeable cation channels are ubiquitously expressed in numerous cell types including macrophages (1). Macrophages express at least 3 different TRP channels, and the properly balanced activation of all these channels together is needed for normal macrophage function. Deletion of any of these channels results in impaired macrophage function and increased susceptibility to infection. Because several of these TRP channels on macrophages are temperature sensitive, they may comprise the link for hypothermia-related infectious complications in trauma, and to a lesser degree, in elective surgical patients. The results show that hypothermia induces effects on monocytes through TRPA1 activation (16). Thermosensitive TRP ion channels such as TRPA1 of sensory fibers influence immune processes. Temperature lowering induces TRPA1 channel activation and significantly stimulates antibody formation in the spleen. Therefore, global loss of TRPA1 expression in the immune system can augment losses in immune cell competence to inhibit infection (17). TRPV4 is associated with transduction of moderate temperature, regulatory volume decrease (RVD) and regulation of stemness (1). TRPV4 is a key regulator of mechanically evoked cellular volume regulation, barrier permeability and immune interactions in lung, bladder, esophagus, skin, gut, kidney, choroid plexus, retinal pigment, and ciliary body epithelia. TRPV4-mediated calcium influx has been linked to cytoskeletal reorganization, cell adhesion, formation of focal adhesions, and adenosine triphosphate (ATP)-dependent modulation of afferent excitation in epithelia. Studies that are relevant are those targeted towards delineating the involvement of TRPV4 channel activation in mediating corneal pain since it was shown that the TRPV4-hemichannel-ATP signaling axis might modulate corneal pain induced by excessive mechanical, osmotic, and chemical stimulation.

## TRP Channel Involvement in Pathobiology of the Skin

TRP channels are expressed in the skin in keratinocytes, sensory neurons, melanocytes, and immune/inflammatory cells in epidermis, dermis, and hypodermis. They maintain skin homeostasis through mediating physiological processes that include sensation. Both excessive or deficient TRP channel activity can induce pathological conditions that arise from chronic pain and itch, dermatitis, vitiligo, alopecia, wound healing, skin carcinogenesis, and skin barrier compromise. Such insight has prompted suggestions that altering TRP channel functionality might provide therapeutic options for ameliorating disorders of sensation, cutaneous homeostasis, and cancer. In normal wound healing induced by injury. Fibrosis subsequent to the self-limiting inflammatory phase hastens the wound closure. It entails increases in collagen deposition, which is essential to restore normal tissue structural integrity. The healing process involves a complex interplay between the coagulation response, innate and adaptive immune reactions, and activation of fibroblasts. Their combined effects underlie regeneration of a normal tissue framework or replacement with a fibrous scar. Furthermore, coactivation of TRPV1 and TRPA1 channels induces chronic inflammatory skin disease such as rosacea, which is associated with neurogenic inflammation. These responses are elicited through primary sensory nerve endings releasing both substance P and CGRP (18). Numerous studies indicated that TRPV1 contributes to maintaining the epidermal barrier function, but it can induce dermatitis in rodents. TRPV1-like immunostaining is more pronounced which is accompanied by mRNA expression upregulation in either the epidermis or in several different human skin diseases, including prurigo nodularis (19), rosacea (20), herpes zoster infection and cancer (21).

TRPV4 is expressed in sensory processes and its expression levels decline in premalignant skin lesions as well as basal and squamous cell carcinomas (22). These functions stem from their activation on keratinocytes. While their involvement on neurons is unclear. TRPA1 plays essential roles in mediating pain, acute and chronic itch perception. Genetic elimination of functional expression of either TRPA1 or TRPV1 on sensory neurons, reduced IL31-induced itch (23). Besides TRPA1 involvement in inducing pathological processes related to itch and pain, its activation also contributes to eliciting inflammation. Specifically, in a acetone-ether-water chronic dry skin model (24) and also in several different models of contact hypersensitivity (25), the mice with diminished TRPA1 expression levels had thinner epidermal layers and lower levels of proinflammatory cytokine expression levels.

## Corneal structures

The maintenance of the tissue integrity of the cornea and the skin is critical for providing protection against pathogenic infiltration. They enable this function through intrinsic mechanisms that overcome a wide range of external threats. Such capability is attributable to their ability to mediate rapid restoration of tissue integrity and organ-specific function. The lacrimal gland secretes the tear film which contains numerous biologically active growth factors that stimulate corneal epithelial cellular proliferation, migration, differentiation, and survival. One of them is epidermal growth factor (EGF), which contributes to promoting epithelial regeneration (26). Beneath the topmost superficial corneal epithelial cell layer is an intermediate layer composed of 2–3 suprabasal wing-shaped cell layers. Beneath this layer is the bottom basal proliferating cells. In this process, proliferating basal cells move upwards and continuously replace the wing cells and superficial cells that are ultimately shed into the tears following undergoing flattening and terminal differentiation. Like in the skin, various collagen types (IV, VII, XII, XV, XVII, XVIII), heparin sulfate proteoglycans, fibronectin, laminins and nidogens are present in the corneal epithelial basement membrane. It is noteworthy that the component makeup of this membrane is heterogenous since its composition varies between the limbal region and the central regions above Bowman’s layer (27–29). Furthermore, its makeup undergoes time dependent changes during postnatal development.

The cornea and the sclera form together the outer shell of the eyeball. Normal vision is dependent on the maintenance of corneal transparency and an optically smooth curvature at the outer surface. The stroma accounts for up to about 90% of the corneal full thickness in between the outer limiting epithelial and inner limiting endothelial layers. It is a layer of avascular connective tissue composed of collagen lamellae interspersed within an extracellular matrix containing different proteoglycans. Other constituent tissues, in addition to the epithelium and its underlying basement membrane, include Bowman’s membrane, stroma, Descemet’s membrane, and the endothelium. Modulation of the stromal collagenous fiber diameter and its structural organization fixed in a geometric array containing keratocytes are critical factors in the maintenance of corneal transparency. The sensory innervation of the eye originates at the trigeminal ganglion. About 70% of the corneal sensory neuron axons are polymodal nociceptors. Sensory nerves are mainly detected in the anterior two thirds of the stromal thickness. The high level of corneal innervation accounts for a sensitivity that is 300 to 400 times greater than that in the skin. Dense nerve endings of numerous nerve fibers form networks at various levels within the tissue (30–35). The thinly myelinated peripheral axons that constitute about 15-20% of the innervation are the only nerves that respond to mechanical forces whose order of magnitude is close to that required to damage corneal epithelial cells. A cold-sensitive thermal receptor group of nerve fibers that compose 10-15% of the total neuronal population possess A-delta and C fibers, which are transiently silenced on warming (36–39). The maintenance of corneal anatomical integrity and function is dependent on sensory nerve activity. Such control is essential since losses in corneal sensory function impair corneal epithelial function and tissue vitality as a consequence of declines in both energy conserving metabolic activity and epithelial cell proliferation. Such defects can in turn compromise the corneal barrier function, which increases the likelihood of pathogenic infiltration into the stroma and the development of neurotrophic keratopathy (40, 41).

## Healing Process of Corneal Wounds

There is much interest amongst basic scientists and clinicians in clarifying the mechanisms underlying corneal wound healing since this tissue is exposed to numerous environmental challenges that can compromise tissue transparency. A very complex and highly orchestrated sequelae mediated by cytokines, growth factors, and chemokines underlie the wound healing response to compromise of tissue structural intactness. These responses stem from an integrated series of events involving epithelial-stromal-neural-lacrimal gland-immune cellular interactions interwoven with the corneal response to injury. Such effects orchestrate the corneal wound healing response and contribute to both restoration of the corneal anatomy and function as well as maintenance of its homeostasis. Corneal disorders are encountered in a clinical setting, that can delay the healing process. The treatment of these disturbances is somewhat limited in their efficacy because it is merely directed towards providing symptomatic relief. Improved therapeutic measures requires gaining insight into the underlying causes of delayed epithelial wound healing. This endeavor necessitates delineating how changes in environmental factors affect corneal epithelial function. An alkali burn model is one of the wound healing models designed to elucidate factors that affect changes in wound healing outcome is. Such an injury induces inflammatory/fibrosis, neovascularization, and opacification resulting in losses in transparency followed by disruption of visual acuity. On the other hand, in a less severe self-limiting wound healing response to a less severe tissue injury, fibrosis facilitates restoration of tissue integrity, which follows a self-limiting acute inflammatory phase. These events are necessary for reversing declines in a well-organized collagenous framework that intersperses keratocyte distribution in a well-defined extracellular matrix (ECM).

Even though there are therapeutic options that provide symptomatic relief from pain and edema, as well as reduce losses in corneal transparency in a clinical setting, they cannot inhibit fibrosis. Another limitation is that they can induce side effects and even toxicity. The cornea usually does not respond to non-penetrating wounds and increases in neovascularizing agents that are not large enough to induce increases in blood and/or lymphatic vessel infiltration. If these maladaptive reactions are induced, they would interfere with restoration of normal vision. Therefore, preserving the avascular state of the cornea depends on sustaining what is referred to as corneal angiogenic privilege. The maintenance of this condition depends on the expression by this tissue of soluble forms of the three major vascular endothelial growth factor (VEGF) receptors. They are all assumed to act as decoy receptors to trap the key angiogenic growth factors VEGF-A, VEGF-C and VEGF-D. Such entrapment is thought to contribute to maintaining corneal avascularity (42–44). During the end of the epithelial healing response, hemidesmosomes are generated, which anchor the newly formed cells to their underlying layers (45, 46). In the skin, there are key factors whose upregulation mediate the wound healing response to injury. They include epidermal growth factor (EGF), transforming growth factor β (TGFβ), hepatocyte growth factor (HGF) and keratinocyte growth factor (KGF) (47). Furthermore, epitheliotropic factors are provided by corneal nerves. They include substance P, calcitonin gene-related peptide (CGRP) and nerve growth factor (NGF), amongst others (34, 48–52). Herpetic disease or systemic diseases such as diabetic polyneuropathy can induce neurotrophic keratopathy, which frequently delays epithelial wound healing. Corneal perforation can arise from severe injury and it may induce persistent and potentially sight-threatening epithelial wounds. Epithelial wound healing is known to be hastened through topical application of recombinant NGF (52, 53). This favorable outcome is the basis for the ongoing clinical trials setup to evaluate its efficacy in the treatment of several diseases of the ocular surface. These conditions include dry eye disease and neurotrophic keratitis. Furthermore, its therapeutic benefit is being evaluated in promoting corneal nerve regeneration after refractive and cataract surgery. However, the mechanism is still not understood that accounts for how NGF eye drops induce a beneficial response to this disease. Besides NGF, EGF, fibroblast growth factor (FGF), and insulin-like growth factor (IGF), have also entered clinical trials with promising results (54–56).

Following epithelial damage, stromal keratocyte apoptosis is the first wound healing response that develops beneath the wound. It is assumed that damaged epithelial cells release cytokines that include: IL-1α, IL-1β, Fas ligand and TNFα (57–61). Initially, keratocyte death renders the area beneath the wound site virtually devoid of all cells. Subsequently, fibroblasts derived from keratocytes proliferate and migrate from the periphery into the wound site. Several growth factors, including TGFβ, platelet-derived growth factor (PDGF), FGF-2 and EGF undergo upregulation during wound healing. During stromal healing, fibroblast migration and activation are assumed to be mediated by (62–65). TGFβ and PDGF. They have also been identified as the main growth factors involved in inducing to undergo fibroblast transdifferentiation into myofibroblasts, which occurs in stromal wounds (66–68). α-Smooth muscle actin (αSMA) upregulation is a definitive biomarker of myofibroblast generation. This response induces wound closure and contraction (69, 70). Other biomarkers of myofibroblast formation include upregulation of vimentin and desmin, which reduces tissue transparency. These changes in growth factor expression patterns are in marked contrast to the physiological milieu sustaining quiescent keratocyte behavior (71, 72).

## TRP Involvement in Mediating Corneal Wound Healing

Our studies are focused on elucidating the roles of different TRP isoforms in mediating wound healing in different injury models induced by perforation, neovascularization, and an epithelial defect inmice. The role was elucidated of TRPV1 cation channel receptor activation in mediating primary repair in an incision-wounded mouse cornea. Selective stimulation of TGFβ1 by stromal incision activated TRPV1, which induced in turn granulation tissue formation (73).

The roles of transient receptor potential TRPV1 and TRPA1 expression were determined on the development of injury-induced neovascularization in the corneal stroma in mice Their involvement was evaluated based on comparing the individual effects of loss of their function on this response. Immunohistochemistry and immunoreactivity detected the active forms of TGFβ1 and VEGF in the stroma at the site of cauterization in mice lacking either TRPV1 or TRPA1 functional expression. However, the losses in TGFβ1 and VEGF immunoreactivity seemed less marked in mice lacking TRPV1 (74). On the other hand, loss of TRPA1 expression suppressed more markedly both stromal neovascularization and inhibited macrophage infiltration. The roles of TRPA1 in mediating injury-induced neovascularization was confirmed by showing that the increases in both VEGF-A and TGF-β1 mRNA expression levels were blunted more in TRPA1 than TRPV1 KO mice. However, in the macrophages their levels were invariant, and their infiltration was inhibited. TRPA1 signaling activation induced by injury may in turn inhibit VEGF-induced neovascularization (75).

## Roles of TRPV1 and TRPV4 in Reversing Corneal Epithelial Defects

Following epithelial debridement, corneal epithelial wound closure was delayed in TRPV1 and TRPV4 KO mice. Such retardation was attributed to lower levels of proinflammatory cytokine expression levels in the TRPV1 and TRPV4 KO than those in the WT mice. Also, neurotrophic keratopathy is one of the refractory corneal disorders that results from damage to the trigeminal nerves and subsequent losses in corneal sensation. This condition precedes various types of corneal disorders, including superficial keratopathy, persistent epithelial defects, and corneal ulcers (41, 76–78). Disorders or abnormal repair of the tissue resulting from impaired sensory innervation or function may impair restoration of corneal epithelial homeostasis and promote instead persistent neurotrophic keratopathy (79–81).

The absence of a suitable experimental mouse model may account for the lack of an effective therapy for neurotrophic keratopathy. To deal with this uncertainty, a stereotactic procedure was used to damage and reduce the function of the first branch of the trigeminal nerve in mice. The damage established a mouse model of neurotrophic keratopathy that simulates the condition described in clinical cases. Under normal conditions, this mouse model possesses an unaltered corneal appearance lacking any tissue inflammation and opacification. However, sensory denervation altered the epithelial debridement induced wound healing response was impaired due to declines in stemness and cell proliferation of the peripheral limbal epithelium. The role was confirmed of TRPV4 function in supporting epithelial wound healing by showing that TRPV4 transfection of a damaged trigeminal nerve rescued the impaired epithelial wound healing response. Partial recovery of the expression levels of the stem/progenitor cell markers, cell proliferation and NGF upregulation in the peripheral limbal epithelium documented the reversal role of TRPV4 transfection. However, TRPV4 gene transfection failed to promote nerve fiber regeneration. Nevertheless, these results show that TRPV4 sensory function is essential to sustain the stem-like peripheral/limbal cell phenotype that is the predominant mechanism underlying corneal epithelial homeostasis.

## Corneal Alkali Burn

One of our areas of interest deals with characterizing how an alkali burn induces corneal fibrosis in mice (15, 82, 83). In our initial study dealing with this question TRPV1 knockdown suppressed a profound decline in corneal transparency. Our initial study dealing with this question showed that the loss of TRPV1 function blocked injury-induced profound increases in inflammation and fibrosis. This suppression suggested that blocking chemical-induced TRPV1 channel activation could provide a therapeutic approach in lessening inflammation-based corneal diseases. We resolved that the wound healing response to an alkali injury stems from TRPV1 activation on resident stromal cells rather than on infiltrating inflammatory cells. This was done by transplanting bone marrow (BM) cells *via* tail vein infusion into recipient mice that had received whole-body irradiation of 12 Gy before BM transfer (i.e. BMT) (from WT mice to KO mice or vice versa). The results that were supportive of this notion showed that TRPV1 KO mice receiving WT BM still had a better wound healing outcome because they had both less inflammation and fibrosis than their WT counterpart chimeras who received instead BMT from KO mice (82).

**Figure 1:**
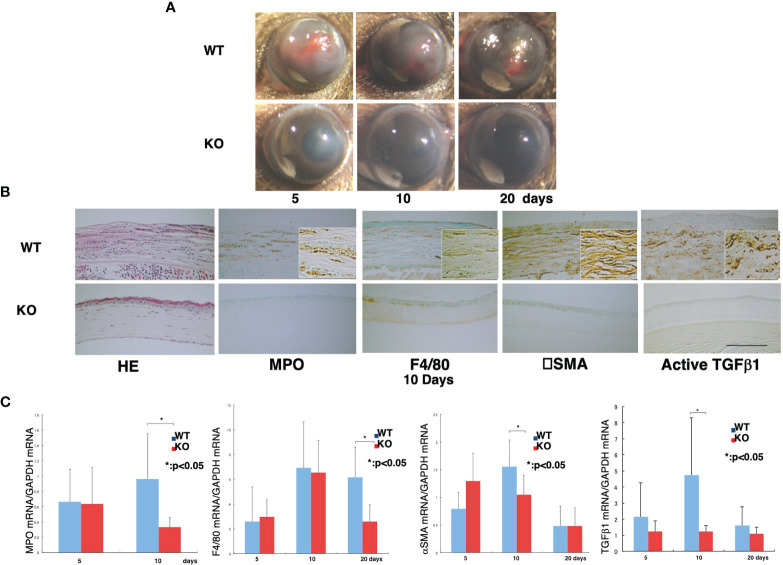
Loss of TRPA1 function blunts inflammatory fibrosis responses to corneal injury.**(A)** Comparison of wound healing progression in wild-type (WT) and TRPA1–null (KO) alkali-burned mouse corneas. At 5, 10, and 20 days after injury, the incidence and degree of opacification and surface irregularity in the healing cornea was more prominent in the WT mice than the TRPA1 KO mice. **(B)** Hematoxylin and eosin (H&E) histological and immunohistochemical changes in burned corneas at day 10. H&E staining shows that the burned corneas have a larger cell population (presumably inflammatory cells) and the WT cornea appear more disorganized than their KO counterpart. The stromal thickness of the WT corneas is greater than in the KO corneas throughout the entire healing period. Immunohistochemistry findings suggest that the density of the myeloperoxidase (MPO)-labeled polymorphonuclear leukocytes (PMNs) and F4/80-labeled macrophages is larger in the WT cornea than in the KO cornea. During the wound healing process, there appear to be many more αPO)-labeled-positive myofibroblasts in the WT mice than in the TRPA1 KO. Furthermore, the anti-αpositive myofibroblasts in the WT mice than in the TRPA1 KO. Furthermore, the anti-rger in the WT **(C)** Active TGFβpositive myofibroblasts in the WT mice than in the TRPA1 KO. Furthermore, the anti-rger in **(C)** Real-time RT-PCR analyses of MPO, F4/80, han in the TRPA1 KO. Furthermore, the anti-rger in the WT c. Activepoint in the TRPA1 KO corneas than those in the WT corneas. Data represent mean ± SEM from five specimens in each condition (bar). *P < 0.05. Reprinted from Okada et al with permission from Lab Invest.

**Figure 2:**
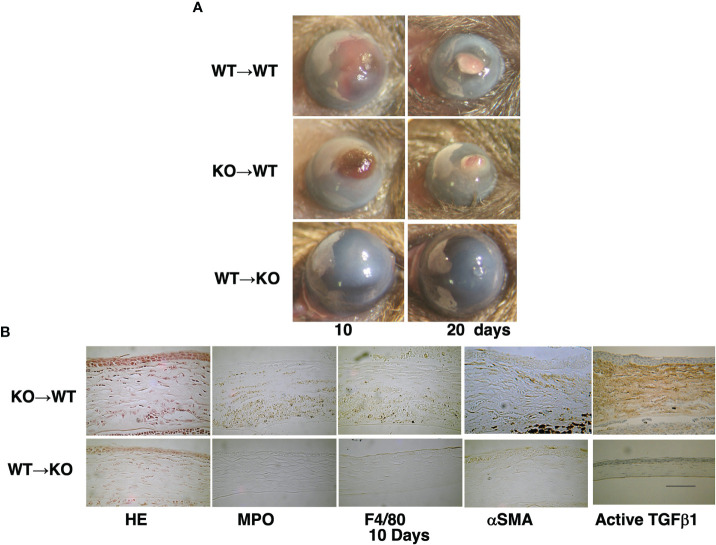
Time dependent healing of an alkali-burned cornea in a bone marrow transplanted (BMT) mouse. **(A)** A wild-type (WT) mouse is injected with bone marrow (BM) from a WT mouse (WT -to-WT) at 10 and 20 days after alkali burning. The responses are compared with those occurring in a WT mouse that receives instead BM from a TRPA1-null (KO) mouse (KO -to-WT). This manipulation results in greater opacification and neovascularization than in the KO mouse that receives instead BM from a WT mouse (WT –to-KO) group. **(B)** H&E histology shows increases in cell density in the swollen stroma of a KO-to-WT cornea as compared with a WT-to-KO tissue. Immunohistochemistry indicates that the cornea of a WT-to-KO mouse has less stromal immunoreactivity as compared with the KO-to-WT tissue. Scale bar, 100 μimmunoreactivity as compared with the KO-to-WT tissuLab Invest.

We also showed the loss of TRPA1-induced signaling inhibits chemical injury-induced corneal inflammation and fibrosis in mice (**Figure 1**). These blunting effects resulting from the loss of TRPA1 function may be a consequence of a lack of TRPA1-induced upregulation of TGFβ1-related signaling cascades in stromal keratocytes or fibroblasts. This difference accompanied decreased activation of Smad3, p38 MAPK, ERK, and JNK. These results suggest that drug-induced TRPA1 inactivation could be of therapeutic value in treating inflammation-based corneal diseases. This conjecture was supported by the findings showing that, the corneal stromal wound healing response was improved in the TRPA1^(-/-)^ knockout (KO) mice. They had more transparent corneas compared with those in the post-alkali burned. wild-type mice. An examination of the corneal surface and eye globes suggested the loss of TRPA1 suppressed post-alkali burn inflammation and fibrosis/scarring, which was confirmed by histology, immunohistochemistry, and gene expression analysis. Therefore, a loss or blocking of TRPA1 activation reduces inflammation and fibrosis/scarring in the corneal stroma during wound healing following an alkali burn in mice. These results suggest that there is crosstalk between the TRPA1- linked signaling pathway axis and its counterpart linked instead to TGFβ R activation by TGFβ. To confirmthat TRPA1 expression on the resident fibroblasts is a critical factor driving fibrosis, we showed that alkali burning in chimera mice receiving reciprocal BMT. TRPA1 KO mice receiving WT BM still exhibited a KO-like phenotype of healing with less inflammatory fibrosis (**Figure 2**). On the other hand, such suppression was absent in the WT counterpart chimeras who received instead BM transplants from TRPA1 KO mice. These effects confirm that the phenotype in a TRPA1 KO mouse is dictated by the expression patterns of inflammatory/fibrotic mediators on resident cells rather than those on the infiltrating inflammatory cells (15).

In TRPV4-null cultured ocular fibroblasts, myofibroblast differentiation was downregulated. IL-6 contributes to inducing this response in WT fibroblasts since injury induced TRPV4 upregulation has a corresponding effect on IL-6 expression levels. Therefore, the lack of IL-6 upregulation in TRPV4-null fibroblasts may account for declines in myofibroblast differentiation. On the other hand, TRPV4 activation induces increases in tissue fibrosis through a signaling pathway that interacts with a TGFβ-dependent signaling pathway in a mouse model of idiopathic lung disease (83). This result differs from the current study in which TRPV4 gene ablation did not alter TGFβ1 signaling events controlling inflammation and fibrosis.

In mouse chimeras, the absence of TRPV4 expression in both corneal resident cells (mesenchymal cells) and infiltrating macrophages accounts for the improved KO healing phenotype. Reciprocal BMT transplants between WT or KO mice who received KO or WT bone marrow, exposure to alkali induced less inflammation in the KO than their WT counterpart (84). As indicated above, the absence of TRPV1, TRPA1 suppresses fibrosis and inflammation through inhibiting transactivation of TGF -induced signaling events whereas in TRPV4 knockout mice. Furthermore, interleukin -6 (IL-6)-induced fibrosis and inflammation depend on stromal TRPV4 access and activation in a severe injury model (82). These undesirable effects in wild-type mice may result in part from enhanced TGF-β1 or IL-6 stimulation of its cognate receptor that augments phospho-p38 MAPK signaling and upregulates both Smad2/3 phosphorylation and a linked cytokine response underlying inflammation and fibrosis. Taken together, injury-induced increases in TRPV1, TRPA1 or TRPV4 activation on resident stromal fibroblasts initially mediates innate immune responses, which provide an adaptive advantage against pathogenic infiltration. However, if this response is dysregulated resulting in persistent rather than self-limiting activation the resulting proinflammatory cytokine storm can lead to blindness.

## TRP Channel Function in Lung, Liver and Intestine

TRPV1, TRPM8, and TRPA1 are different channel subtypes expressed on airway sensory nerve terminals and serve as molecular detectors of thermal and chemical stimuli, their activation induces pain, inflammation, and fibrosis in many organs. They are activated by exogenous ligands such as pepper, mint, and mustard plants and in turn induce acute or persistent pain (85). Allergen-induced asthma stems from TRPA1 upregulation which has a proinflammatory role in the lungs of mice (86). Recently, it was reported that TRPV4 activation is defective and contributes to inducing idiopathic lung fibrosis through promoting fibroblast transdifferentiation into myofibroblasts in the airway epithelia of cystic fibrosis patients and in mice (87, 88).

Furthermore, it was recently shown that TRPV4 expression was upregulated in pulmonary fibrotic tissues and cells (83). In agreement with this finding, TRPV4-deficient mice were found to be protected from fibrosis with less lung collagen accumulation, myofibroblast differentiation and lower mortality. Also, TRP channel activation on sensory neurons has a role in inducing the cough reflex in chronic lung diseases. This realization has prompted suggestions that these ion channels may be appropriate targets to treat such symptomology.

Recently, TRPV4 expression was found to be elevated in hepatic fibrotic tissues and TGF-β1 expression levels were also upregulated in-stimulated hepatic stellate cells (89). Furthermore, TRPV4 was a direct microRNA-203 target, which in turn promoted TGF-β1-induced hepatic stellate cell proliferation (89). Also, TRPA1 is highly expressed in the intestinal lamina propria and in the stenotic intestinal regions of Crohn’s disease (CD) patients, which increased TRPA1/heat shock protein 47 double-positive cells accumulation in the stenotic intestinal regions of CD patients. On the other hand, the anti-inflammatory actions of TRPA1 may protect against intestinal fibrosis. If such an effect is confirmed, drug targeting TRPA1 expression may be provide a novel approach to improve management of highly incurable inflammatory/fibrotic disorders, especially CD.

## Potential Drug Targets

**Figure 3:**
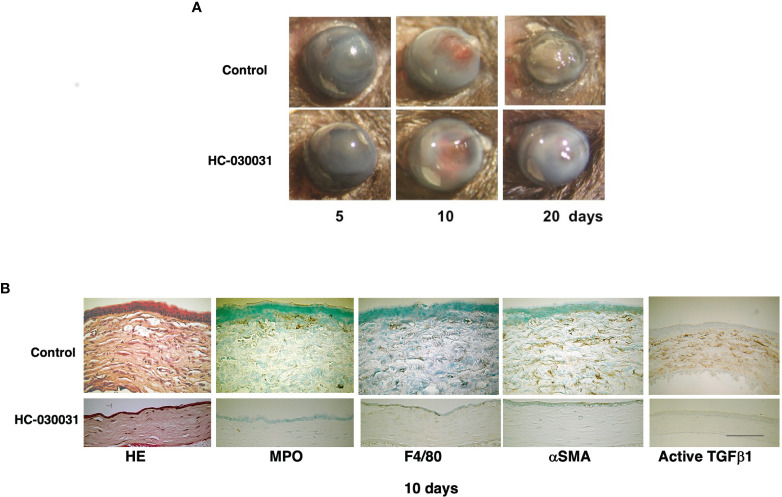
TRPA1 antagonist (HC-030031) treatment improves alkali burn-induced wound healing response in wild-type (WT) mice. **(A)** In WT mice, wound healing progression is compared at 5, 10 or 20 days after injury in the presence and absence of HC-030031. At each time point, corneal transparency restoration is markedly improved in the mice treated with this antagonist. At day 20, corneal transparency is restored in an antagonist treated mouse, but not in an untreated mouse. **(B)** The immunohistochemical staining patterns and the H&E stained histology are shown of burned corneas at day 10. The stromal organization is more poorly preserved in the untreated mice than in the antagonist treated mice. The infiltration levels of MPO-labeled neutrophils and F4/80-positive macrophages are lower in the antagonist treated mice than in the untreated counterpart. Similarly, the αhe immunohistochemicastaining are also less in the antagonist treated cornea than in their untreated counterpart. Scale bar, 100 Lab Invest.

The protective role is still not clear of TRP expression in maintaining corneal homeostasis by offsetting the damaging effects of environmental stresses on tissue function. On the other hand, their involvement in inducing corneal pathogenesis requires clarification. Our studies identified TRPV1, TRPA1 and TRPV4 as potential drug targets to treat sight compromising corneal injuries caused by a chemical burn in mice (15, 82, 83) (**Figure 3**). Their individual roles were clarified based on showing that severe injury of gene silenced littermates induced chronic immune responses and stromal fibrosis that were all remarkably attenuated resulting in hastening of a much more favorable wound healing outcome. However, in these gene silenced mice, the wound healing response to epithelial defects was delayed because transient activation of these TRPs subtypes in wildtype mice promotes cell proliferation and migration. These diametrically opposed effects on the time required for completion of the wound healing response hinders designing drugs, which can selectively activate epithelial TRPV1 and TRPV4 channels to accelerate re-epithelialization, but at the same time inhibit stromal TRPV1, TRPA1 and TRPV4 activation and suppress chronic inflammation as well as fibrosis. Overcoming this complication depends on determining if it is possible to design agents that can target specific sites that solely modulate a response of interest. In other words, meaningful drug development awaits delineating whether there are specific sites on TRP channel structure that elicit each of these responses. Some progress has been reported in this regard by identifying site specific domains on TRP channels whose occupancy induces a specific response. Specifically, it is now possible with different novel TRPV1 antagonists to selectively inhibit pain induction without inducing a rise in body temperature. Unlike these novel antagonists, which are currently being evaluated for possible clinical use, systemic inhibition of TRP channel function with more extensively characterized antagonists causes body temperature to rise even though they inhibit inflammation and myofibroblast transdifferentiation.

**Figure 4:**
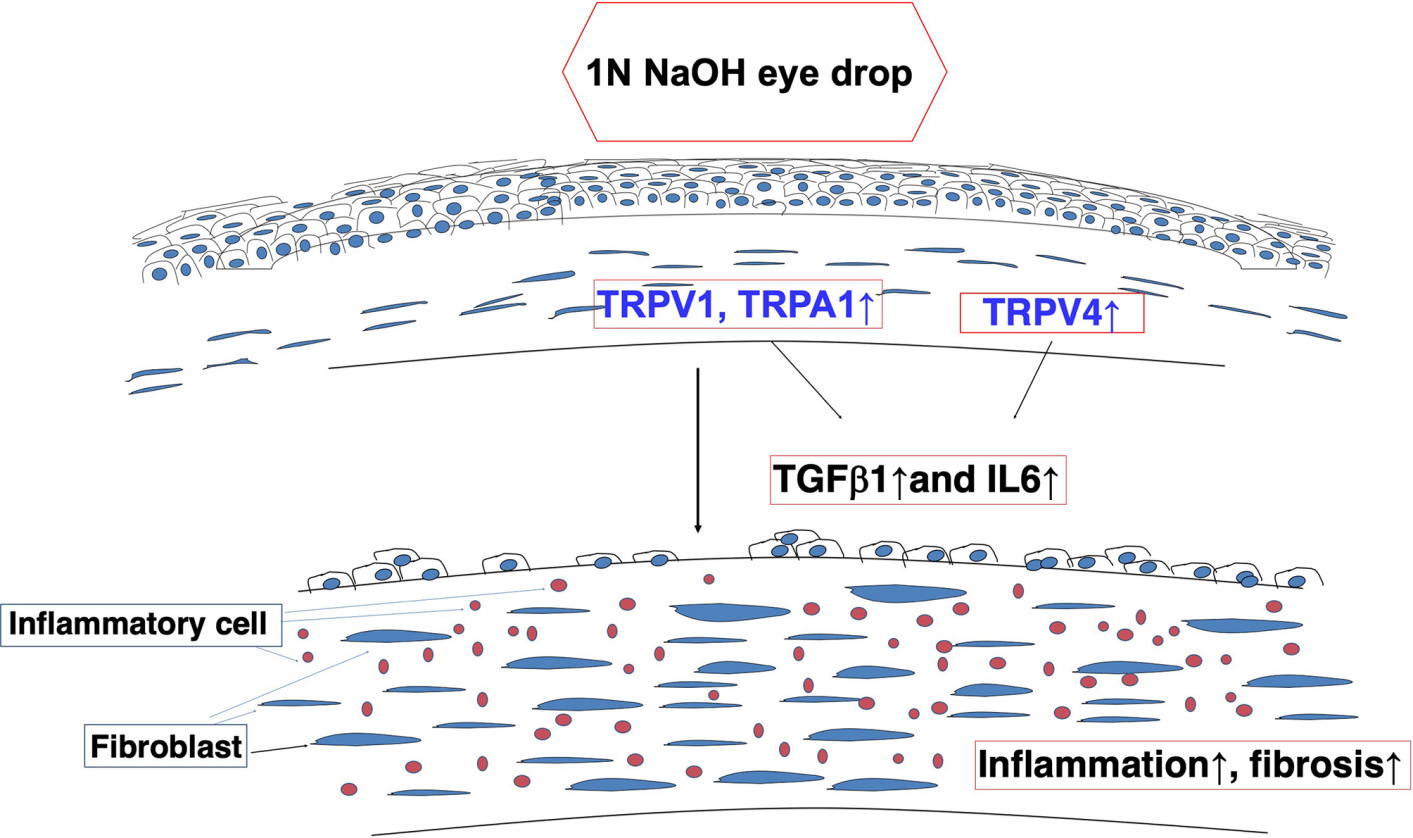
Alkali burn injury activates TRP signaling and induces inflammatory/fibrogenic responses in mouse corneas. TRPV1 and TRPA1 transactivation of transforming growth factor (TGF)β1 _-_linked signaling induces fibroblasts to elicit fibrosis and inflammation - Interleukin-6 (IL-6)-induced fibrosis and inflammation are dependent on TRPV4 expression and activation in a severe injury model.

The roles of systemic TRPV1, TRPA1 or TRPV4 channels in mediating control of functions related to corneal wound healing were confirmed based on characterizing the effects of loss of function on the responses underlying injury-induced corneal wound healing. With TRPV1 and TRPA1, it was possible to document drug selectivity based on showing that antagonist administration had effects that were similar to those induced in each of the knockout models. Furthermore, our results suggest that each of the signaling pathways linked to TRPV1, TRPA1 and TRPV4 activation interact with one another during the wound healing process. Finally, such interactions can also involve crosstalk with signaling pathways mediating IL-6 and TGFβ control of responses that underlie the development of inflammatory/fibrosis (**Figure 4**).

## Author Contributions

YO: Did the research and wrote this paper. TS: Helped with the research. PR: Wrote and edited the paper. SS: Invented the research and edit the paper. All authors contributed to the article and approved the submitted version.

## Funding

This study was supported by the Grants from the Ministry of Education, Science, Sports and Culture of Japan (C21592241 to YO, C40433362 to TS, C19592036 to SS).

## Conflict of Interest

The authors declare that the research was conducted in the absence of any commercial or financial relationships that could be construed as a potential conflict of interest.

## Publisher’s Note

All claims expressed in this article are solely those of the authors and do not necessarily represent those of their affiliated organizations, or those of the publisher, the editors and the reviewers. Any product that may be evaluated in this article, or claim that may be made by its manufacturer, is not guaranteed or endorsed by the publisher.

## References

[1] Lapajne L Lakk M Yarishkin O Gubeljak L Hawlina M Križaj D. Polymodal Sensory Transduction in Mouse Corneal Epithelial Cells. Invest Ophthalmol Vis Sci (2020) 61:2. doi: 10.1167/iovs.61.4.2 PMC7401707 32271891

[2] Sousa-Valente J Brain SD. A Historical Perspective on the Role of Sensory Nerves in Neurogenic Inflammation. Semin Immunopathol (2018) 40:229–36. doi: 10.1007/s00281-018-0673-1 PMC5960476 29616309

[3] Montell C Rubin GM. Molecular Characterization of the Drosophila Trp Locus: A Putative Integral Membrane Protein Required for Phototransduction. Neuron (1989) 2:1313–23. doi: 10.1016/0896-6273(89)90069-X 2516726

[4] Minke B. The History of the Drosophila TRP Channel: The Birth of a New Channel Superfamily. J Neurogenet (2010) 24:216–33. doi: 10.3109/01677063.2010.514369 PMC3103766 21067449

[5] Owsianik G Talavera K Voets T Nilius B. Permeation and Selectivity of TRP Channels. Annu Rev Physiol (2006) 68:685–717. doi: 10.1146/annurev.physiol.68.040204.101406 16460288

[6] Pedersen SF Owsianik G Nilius B. TRP Channels: An Overview. Cell Calcium (2005) 38:233–52. doi: 10.1016/j.ceca.2005.06.028 16098585

[7] Ramsey IS Delling M Clapham DE. An Introduction to TRP Channels. Annu Rev Physiol (2006) 68:619–47. doi: 10.1146/annurev.physiol.68.040204.100431 16460286

[8] Adcock JJ. TRPV1 Receptors in Sensitisation of Cough and Pain Reflexes. Pulm Pharmacol Ther (2009) 22:65–70. doi: 10.1016/j.pupt.2008.12.014 19141328

[9] Ahern GP. Activation of TRPV1 by the Satiety Factor Oleoylethanolamide. J Biol Chem (2003) 278:30429–34. doi: 10.1074/jbc.M305051200 12761211

[10] Yang F Zheng J. Understand Spiciness: Mechanism of TRPV1 Channel Activation by Capsaicin. Protein Cell (2017) 8:169–77. doi: 10.1007/s13238-016-0353-7 PMC5326624 28044278

[11] Yang Y Yang H Wang Z Mergler S Wolosin JM. Reinach PS Functional TRPV1 Expression in Human Corneal Fibroblasts. Exp Eye Res (2013) 107:121–9. doi: 10.1016/j.exer.2012.11.004 PMC3556194 23232207

[12] Yap JMG Ueda T Takeda N Fukumitsu K Fukuda S Uemura T. An Inflammatory Stimulus Sensitizes TRPA1 Channel to Increase Cytokine Release in Human Lung Fibroblasts. Cytokine (2020) 129:155027. doi: 10.1016/j.cyto.2020.155027 32050145

[13] Büch TR Schäfer EA Demmel MT Boekhoff I Thiermann H Gudermann T. Functional Expression of the Transient Receptor Potential Channel TRPA1, a Sensor for Toxic Lung Inhalants, in Pulmonary Epithelial Cells. Chem Biol Interact (2013) 206(3):462–71. doi: 10.1016/j.cbi.2013.08.012 23994502

[14] Staruschenko A Jeske NA Akopian AN. Contribution of TRPV1-TRPA1 Interaction to the Single Channel Properties of the TRPA1 Channel. J Biol Chem (2010) 285:15167–77. doi: 10.1074/jbc.M110.106153 PMC2865321 20231274

[15] Okada Y Shirai K Reinach PS Kitano-Izutani A Miyajima M Flanders KC. TRPA1 Is Required for TGF-b Signaling and Its Loss Blocks Inflammatory Fibrosis in Mouse Corneal Stroma. Lab Invest (2014) 94:1030–41. doi: 10.1038/labinvest.2014.85 PMC5919187 25068659

[16] Billeter AT Galbraith N Walker S Lawson C Gardner SA Sarojini H. TRPA1 Mediates the Effects of Hypothermia on the Monocyte Inflammatory Response. Surgery (2015) 158:646–54. doi: 10.1016/j.surg.2015.03.065 26054320

[17] Kozyreva TV Khramova GM. Effects of Activation of Skin Ion Channels TRPM8, TRPV1, and TRPA1 on the Immune Response. Comparison With Effects of Cold and Heat Exposure. J Therm Biol (2020) 93:102729. doi: 10.1016/j.jtherbio.2020.102729 33077140

[18] Steinhoff M Schauber J Leyden JJ. New Insights Into Rosacea Pathophysiology: A Review of Recent Findings. J Am Acad Dermatol (2013) 69:S15–26. doi: 10.1016/j.jaad.2013.04.045 24229632

[19] Stander S Moormann C Schumacher M Buddenkotte J Artuc M Shpacovitch V. Expression of Vanilloid Receptor Subtype 1 in Cutaneous Sensory Nerve Fibers, Mast Cells, and Epithelial Cells of Appendage Structures. Exp Dermatol (2004) 13:129–39. doi: 10.1111/j.0906-6705.2004.0178.x 14987252

[20] Sulk M Seeliger S Aubert J Schwab VD Cevikbas F Rivier M. Distribution and Expression of Non-Neuronal Transient Receptor Potential (TRPV) Ion Channels in Rosacea. J Investig Dermatol (2012) 132:1253–62. doi: 10.1038/jid.2011.424 PMC3305847 22189789

[21] Han SB Kim H Cho SH Lee JD Chung JH Kim HS. Transient Receptor Potential Vanilloid-1 in Epidermal Keratinocytes may Contribute to Acute Pain in Herpes Zoster. Acta Derm Venereol (2016) 96:319–22. doi: 10.2340/00015555-2247 26390894

[22] Fusi C Materazzi S Minocci D Maio V Oranges T Massi D. Transient Receptor Potential Vanilloid 4 (TRPV4) Is Downregulated in Keratinocytes in Human Non-Melanoma Skin Cancer. J Investig Dermatol (2014) 134:2408–17. doi: 10.1038/jid.2014.145 24643128

[23] Cevikbas F Wang X Akiyama T Kempkes C Savinko T Antal A. A Sensory Neuron-Expressed IL-31 Receptor Mediates T Helper Cell-Dependent Itch: Involvement of TRPV1 and TRPA1. J Allergy Clin Immunol (2014) 133:448–60. doi: 10.1016/j.jaci.2013.10.048 PMC3960328 24373353

[24] Wilson SR Nelson AM Batia L Morita T Estandian D Owens DM. The Ion Channel TRPA1 Is Required for Chronic Itch. J Neurosci (2013) 33:9283–94. doi: 10.1523/JNEUROSCI.5318-12.2013 PMC3752436 23719797

[25] Liu B Escalera J Balakrishna S Fan L Caceres AI Robinson E. TRPA1 Controls Inflammation and Pruritogen Responses in Allergic Contact Dermatitis. FASEB J (2013) 27:3549–63. doi: 10.1096/fj.13-229948 PMC3752543 23722916

[26] Klenkler B Sheardown H Jones L. Growth Factors in the Tear Film: Role in Tissue Maintenance, Wound Healing, and Ocular Pathology. Ocul Surf (2007) 5:228–39. doi: 10.1016/S1542-0124(12)70613-4 17660896

[27] Kabosova A Azar DT Bannikov GA Campbell KP Durbeej M Ghohestani RF. Compositional Differences Between Infant and Adult Human Corneal Basement Membranes. Invest Ophthalmol Vis Sci (2007) 48:4989–99. doi: 10.1167/iovs.07-0654 PMC2151758 17962449

[28] Herwig MC Müller AM Holz FG Loeffler KU. Immunolocalization of Different Collagens in the Cornea of Human Fetal Eyes: A Developmental Approach. Curr Eye Res (2013) 38:60–9. doi: 10.3109/02713683.2012.738461 23130612

[29] Torricelli AAM Singh V Santhiago MR Wilson SE. The Corneal Epithelial Basement Membrane: Structure, Function, and Disease. Invest Ophthalmol Vis Sci (2013) 54:6390–400. doi: 10.1167/iovs.13-12547 PMC3787659 24078382

[30] Tervo T Palkama A. Innervation of the Rabbit Cornea. A Histochemical and of Ocular Surface. Ann Med (1978) 24:19–27. doi: 10.3109/07853899209164141 685646

[31] Marfurt CF Kingsley RE Fchtenkamp SE. Sebsory and Sympathetic Innervation of the Mammalian Cornea. A Retrograde Tracing Study. Invest Ophthalmol Vis Sci (1989) 30:461–72. 2494126

[32] Muller LJ Pels L Vrensen GF. Ultrastructural Organization of Human Corneal Nerves. Invest Ophthalmol Vis Sci (1996) 37:476–88. 8595948

[33] Muller LJ Vrensen GF Pels L Cardozo BN Willekens B. Architecture of Human Corneal Nerves. Invest Ophthalmol Vis Sci (1997) 38:985–94. 9112994

[34] Muller LJ Marfurt CF Kruse F Tervo TM. Corneal Nerves: Structure, Contents and Function. Exp Eye Res (2003) 76:525–42. doi: 10.1016/S0014-4835(03)00050-2 12697417

[35] Marfurt CF Murphy CJ Florczak L. Morphology and Neurochemistry of Canine Corneal Innervation. Invest Ophthalmol Vis Sci (2001) 42:2242–51. 11527937

[36] Brock JA McLachlan EM Belmonte C. Tetrodotoxin-Resistant Impulses in Single Nociceptor Nerve Terminal in Guinea-Pig Cornea. J Physiol (1998) 512:211–7. doi: 10.1111/j.1469-7793.1998.211bf.x PMC2231175 9729630

[37] Tanelian DL Beuerman RW. Responses of Rabbit Corneal Nociceptors to Mechanical and Thermal Stimulation. Exp Neurol (1984) 84:165–78. doi: 10.1016/0014-4886(84)90013-X 6705882

[38] Jaffe M. Neuroparalytic Keratitis. Arch Ophthalmol (1938) 20:688–9.

[39] Pannabecker CL. Keratitis Neuroparalica. Arch Ophthalmol (1944) 32:456–63. doi: 10.1001/archopht.1944.00890120036003

[40] Sigelman S Friedenwald JS. Mitotic and Wound-Healing Activities of the Corneal Epithelium. Arch Ophthalmol (1954) 53:46–57. doi: 10.1001/archopht.1954.00920050048005 13170864

[41] Mishima S. The Effects of the Denervation and the Stimulation of the Sympathetic and Trigeminal Nerve on the Mitotic Rate of the Corneal Epithelium in Rabbit. Jpn J Ophthalmol (1957) 1:65–73.

[42] Albuquerque RJC Hayashi T Cho WG Kleinman ME Dridi S Takeda A. Alternatively Spliced Vascular Endothelial Growth Factor Receptor-2 Is an Essential Endogenous Inhibitor of Lymphatic Vessel Growth. Nat Med (2009) 15:1023–30. doi: 10.1038/nm.2018 PMC2882165 19668192

[43] Ambati BK Nozaki M Singh N Takeda A Jani PD Suthar T. Corneal Avascularity is Due to Soluble VEGF Receptor-1. Nature (2006) 443:993–7. doi: 10.1038/nature05249 PMC2656128 17051153

[44] Singh N Tiem M Watkins R Cho YK Wang Y Olsen T. Soluble Vascular Endothelial Growth Factor Receptor 3 Is Essential for Corneal Alymphaticity. Blood (2013) 121:4242–9. doi: 10.1182/blood-2012-08-453043 PMC3656456 23476047

[45] Zieske JD. Extracellular Matrix and Wound Healing. Curr Opin Ophthalmol (2001) 12:237–41. doi: 10.1097/00055735-200108000-00001 11507335

[46] Buck RC. Hemidesmosomes of Normal and Regenerating Mouse Corneal Epithelium. Virchows Arch B Cell Pathol Incl Mol Pathol (1982) 41:1–16. doi: 10.1007/BF02890267 6134376

[47] Spadea L Giammaria D Trabucco P. Corneal Wound Healing After Laser Vision Correction. Br J Ophthalmol (2016) 100:28–33. doi: 10.1136/bjophthalmol-2015-306770 26405102

[48] Garcia-Hirschfeld J Lopez-Briones LG Belmonte C. Neurotrophic Influences on Corneal Epithelial Cells. Exp Eye Res (1994) 59:597–605. doi: 10.1006/exer.1994.1145 9492761

[49] Reid TW Murphy CJ Iwahashi CK Foster BA Mannis MJ. Stimulation of Epithelial Cell Growth by the Neuropeptide Substance P. J Cell Biochem (1993) 52:476–85. doi: 10.1002/jcb.240520411 7693729

[50] Nakamura M Chikama T Nishida T. Up-Regulation of Integrin α 5 Expression by Combination of Substance P and Insulin-Like Growth Factor-1 in Rabbit Corneal Epithelial Cells. Biochem Biophy Re Commun (1998) 246:777–82. doi: 10.1006/bbrc.1998.8704 9618288

[51] Mikulec AA Tanelian DL. CGRP Increases the Rate of Corneal Re-Epithelialization in an *In Vitro* Whole Mount Preparation. J Ocul Pharmacol Ther (1996) 12:417–23. doi: 10.1089/jop.1996.12.417 8951678

[52] Lambiase A Bonini S Aloe L Rama P Bonini S. Anti-Inflammatory and Healing Properties of Nerve Growth Factor in Immune Corneal Ulcers With Stromal Melting. Arch Ophthalmol (2000) 118:1446–9. doi: 10.1001/archopht.118.10.1446 11030834

[53] Lambiase A Rama P Bonini S Caprioglio G Aloe L. Topical Treatment With Nerve Growth Factor for Corneal Neurotrophic Ulcers. N Engl J Med (1998) 338:1174–80. doi: 10.1056/NEJM199804233381702 9554857

[54] Pastor JC Calonge M. Epidermal Growth Factor and Corneal Wound Healing. A Multicenter Study. Cornea (1992) 11:311–4. doi: 10.1097/00003226-199207000-00007 1424650

[55] Meduri A Aragona P Grenga PL Roszkowska AM. Effect of Basic Fibroblast Growth Factor on Corneal Epithelial Healing After Photorefractive Keratectomy. J Refract Surg (2012) 28:220–3. doi: 10.3928/1081597X-20120103-02 22230058

[56] Yamada N Matsuda R Morishige N Yanai R Chikama T-I Nishida T. Open Clinical Study of Eye-Drops Containing Tetrapeptides Derived From Substance P and Insulin-Like Growth Factor-1 for Treatment of Persistent Corneal Epithelial Defects Associated With Neurotrophic Keratopathy. Br J Ophthalmol (2008) 92:896–900. doi: 10.1136/bjo.2007.130013 18511539

[57] Wilson SE He YG Weng J Li Q McDowall AW Vital M. Epithelial Injury Induces Keratocyte Apoptosis: Hypothesized Role for the Interleukin-1 System in the Modulation of Corneal Tissue Organization and Wound Healing. Exp Eye Res (1996) 62:325–7. doi: 10.1006/exer.1996.0038 8795451

[58] Mohan RR Liang Q Kim WJ Helena MC Baerveldt F Wilson SE. Apoptosis in the Cornea: Further Characterization of Fas/Fas Ligand System. Ex Eye Res (1997) 65:575–89. doi: 10.1006/exer.1997.0371 9464190

[59] Mohan RR Mohan RR Kim WJ Wilson SE. Modulation of TNF-α-Induced Apoptosis in Corneal Fibroblasts by Transcription Factor NF-κb. Invest Ophthalmol Vis Sci (2000) 41:1327–36. 10798647

[60] Ambrósio R Kara-José N Wilson SE. Early Keratocyte Apoptosis After Epithelial Scrape Injury in the Human Cornea. Exp Eye Res (2009) 89:597–9. doi: 10.1016/j.exer.2009.06.003 PMC2743797 19523947

[61] Wilson SE Mohan RR Mohan RR Ambrósio R Hong J Lee J. The Corneal Wound Healing Response: Cytokine-Mediated Interaction of the Epithelium, Stroma, and Inflammatory Cells. Prog Retin Eye Res (2001) 20:625–37. doi: 10.1016/S1350-9462(01)00008-8 11470453

[62] Petroll WM Kivanany PB Hagenasr D Graham EK. Corneal Fibroblast Migration Patterns During Intrastromal Wound Healing Correlate With ECM Structure and Alignment. Invest Ophthalmol Vis Sci (2015) 56:7352–61. doi: 10.1167/iovs.15-17978 PMC4645812 26562169

[63] Andresen JL Ledet T Ehlers N. Keratocyte Migration and Peptide Growth Factors: The Effect of PDGF, βFGF, EGF, IGF-I, αFGF and TGF-β on Human Keratocyte Migration in a Collagen Gel. Curr Eye Res (1997) 16:605–13. doi: 10.1076/ceyr.16.6.605.5081 9192171

[64] Jester JV Barry-Lane PA Petroll WM Olsen DR Cavanagh HD. Inhibition of Corneal Fibrosis by Topical Application of Blocking Antibodies to TGF β in the Rabbit. Cornea (1997) 16:177–87. 9071531

[65] Jester JV Ho-Chang J. Modulation of Cultured Corneal Keratocyte Phenotype by Growth Factors/Cytokines Control *In Vitro* Contractility and Extracellular Matrix Contraction. Exp Eye Res (2003) 77:581–92. doi: 10.1016/S0014-4835(03)00188-X 14550400

[66] Masur SK Dewal HS Dinh TT Erenburg I Petridou S. Myofibroblasts Differentiate From Fibroblasts When Plated at Low Density. Proc Natl Acad Sci USA (1996) 93:4219–23. doi: 10.1073/pnas.93.9.4219 PMC39515 8633044

[67] Jester JV Huang J Barry-Lane PA Kao WW Petroll WM Cavanagh HD. Transforming Growth Factor(β)-Mediated Corneal Myofibroblast Differentiation Requires Actin and Fibronectin Assembly. Invest Ophthalmol Vis Sci (1999) 40:1959–67. 10440249

[68] Jester JV Huang J Petroll WM Cavanagh HD. TGFβ Induced Myofibroblast Differentiation of Rabbit Keratocytes Requires Synergistic TGFβ, PDGF and Integrin Signaling. Exp Eye Res (2002) 75:645–67. doi: 10.1006/exer.2002.2066 12470966

[69] Garana RM Petroll WM Chen WT Herman IM Barry P Andrews P. Radial Keratotomy. II. Role of the Myofibroblast in Corneal Wound Contraction. Invest Ophthalmol Vis Sci (1992) 33:3271–82. 1428702

[70] Jester JV Petroll WM Barry PA Cavanagh HD. Expression of α-Smooth Muscle (α-SM) Actin During Corneal Stromal Wound Healing. Invest Ophthalmol Vis Sci (1995) 36:809–19. 7706029

[71] Jester JV Moller-Pedersen T Huang J Sax CM Kays WT Cavangh HD. The Cellular Basis of Corneal Transparency: Evidence for ‘Corneal Crystallins’. J Cell Sci (1999) 112:613–22. doi: 10.1242/jcs.112.5.613 9973596

[72] Chaurasia SS Kaur H de Medeiros FW Smith SD Wilson SE. Dynamics of the Expression of Intermediate Filaments Vimentin and Desmin During Myofibroblast Differentiation After Corneal Injury. Exp Eye Res (2009) 89:133–9. doi: 10.1016/j.exer.2009.02.022 PMC2716066 19285070

[73] Nidegawa-Saitoh Y Sumioka T Okada Y Reinach PS Flanders KC Liu CY. Impaired Healing of Cornea Incision Injury in a TRPV1-Deficient Mouse. Cell Tissue Res (2018) 374:329–38. doi: 10.1007/s00441-018-2878-y PMC6209059 29971480

[74] Tomoyose K Okada Y Sumioka T Miyajima M Flanders KC Shirai K. Suppression of *In Vivo* Neovascularization by the Loss of TRPV1 in Mouse Cornea. J Ophthalmol (2015) 2015:706404. doi: 10.1155/2015/706404 26491553 PMC4600561

[75] Usui-Kusumoto K Iwanishi H Ichikawa K Okada Y Sumioka T Miyajima M. Suppression of Neovascularization in Corneal Stroma in a TRPA1-Null Mouse. Exp Eye Res (2019) 181:90–7. doi: 10.1016/j.exer.2019.01.002 30633924

[76] Lim CH. Innervation of the Cornea of Monkeys and the Effects of Denervation. Br J Physiol Opt (1976) 31:38–42. 830034

[77] Beuerman RW Schimmelpfenning B. Sensory Denervation of the Rabbit Cornea Affects Epithelial Properities. Exp Neurol (1980) 69:196–201. doi: 10.1016/0014-4886(80)90154-5 7389846

[78] Klyce SD Beuerman RW Crosson CE. Alteration of Corneal Epithelial Ion Transport by Sympathectomy. Invest Ophthalmol Vis Sci (1985) 26:434–42. 2858455

[79] Okada Y Reinach P Kitano A Shirai K Kao WW Saika S. Neurotrophic Keratopathy; its Pathophysiology and Treatment. Histol Histopathol (2010) 25:771–80. doi: 10.14670/HH-25.771 20376784

[80] Sacchetti M Lambiase A. Diagnosis and Management of Neurotrophic Keratitis. Clin Ophthalmol (2014) 8:571–9. doi: 10.2147/OPTH.S45921 PMC3964170 24672223

[81] Bonini S Rama P Olzi D Lambiase A. Neurotrophic Keratitis. Eye (Lond) (2003) 17:989–95. doi: 10.1038/sj.eye.6700616 14631406

[82] Okada Y Reinach PS Shirai K Kitano A Kao WW Flanders KC. TRPV1 Involvement in Inflammatory Tissue Fibrosis in Mice. Am J Pathol (2011) 178:2654–64. doi: 10.1016/j.ajpath.2011.02.043 PMC3124334 21641388

[83] Rahaman SO Grove LM Paruchuri S Southern BD Abraham S Niese KA. TRPV4 Mediates Myofibroblast Differentiation and Pulmonary Fibrosis in Mice. J Clin Invest (2014) 124:5225–38. doi: 10.1172/JCI75331 PMC4348970 25365224

[84] Okada Y Shirai K Miyajima M Reinach PS Yamanaka O Sumioka T. Loss of TRPV4 Function Suppresses Inflammatory Fibrosis Induced by Alkali-Burning Mouse Corneas. PloS One (2016) 11:e0167200. doi: 10.1371/journal.pone.0167200 28030558 PMC5193391

[85] Julius D. TRP Channels and Pain. Annu Rev Cell Dev Biol (2013) 29:355–84. doi: 10.1146/annurev-cellbio-101011-155833 24099085

[86] Caceres AI Brackmann M Elia MD Bessac BF del Camino D D’Amours M. A Sensory Neuronal Ion Channel Essential for Airway Inflammation and Hyperreactivity in Asthma. Proc Natl Acad Sci USA (2009) 106:9099–104. doi: 10.1073/pnas.0900591106 PMC2684498 19458046

[87] Bonvini SJ Belvisi MG. Cough and Airway Disease: The Role of Ion Channels. Pulm Pharmacol Ther (2017) 47:21–8. doi: 10.1016/j.pupt.2017.06.009 28669932

[88] Dietrich A Steinritz D Gudermann T. Transient Receptor Potential (TRP) Channels as Molecular Targets in Lung Toxicology and Associated Diseases. Cell Calcium (2017) 67:123–37. doi: 10.1016/j.ceca.2017.04.005 28499580

[89] Zhan L Yang Y Ma TT Huang C Meng XM Zhang L. Transient Receptor Potential Vanilloid 4 Inhibits Rat HSC-T6 Apoptosis Through Induction of Autophagy. Mol Cell Biochem (2015) 402:9–22. doi: 10.1007/s11010-014-2298-6 25600591

