# Supplementary material for: Roles of Epithelial and Mesenchymal TRP Channels in Mediating Inflammatory Fibrosis
Source: Front Immunol. 2022 Jan 4;12:731674. doi: 10.3389/fimmu.2021.731674 (PMC8763672; doi:10.3389/fimmu.2021.731674)
Supplement: Supplementary file 1 [file Table_1.pdf]

Table 1

	Induce Molekule	Depend on Phenotype
TRPV1	TGFβ1	Resident tissue cells
TRPA1	TGFβ1	Resident tissue cells
TRPV4	IL-6	Resident tissue cells and inflammatory cells
